# Exploring the Multilevel Determinants of Suboptimal Maternal and Child Continuum of Care in Indonesia

**DOI:** 10.1007/s10995-025-04110-w

**Published:** 2025-07-04

**Authors:** Vitri Widyaningsih, Itismita Mohanty, Tri Mulyaningsih, Tesfaye Alemayehu Gebremedhin, Riyana Miranti, Nurussyifa Afiana Zaen, Septyan Dwi Nugroho, Akhmad Azmiardi, Ari Probandari

**Affiliations:** 1 Faculty of Medicine, Universitas Sebelas Maret, Jalan Ir. Sutami 36 A, Kentingan, Jebres, Surakarta, Central Java 57126 Indonesia; 2 Disease Control Research Group, Universitas Sebelas Maret, Surakarta, Central Java Indonesia; 3 Health Research Institute, University of Canberra, Canberra, Australia; 4 Department of Economics, Faculty of Economics and Business, Universitas Sebelas Maret, Surakarta, Central Java Indonesia; 5 Faculty of Business, Government and Law, University of Canberra, Canberra, Australia; 6 School of Oil and Gas Technology of Cilacap, Cilacap, Indonesia; 7 Master of Public Health Study Program, Faculty of Public Health, Mulawarman University, Samarinda, Indonesia

**Keywords:** Maternal and child heath, Maternal continuum of care, Vaccination, Multilevel determinants

## Abstract

**Objective:**

Understanding the multilevel factors associated with completeness of care across the continuum of maternal and child health is needed in order to reduce maternal and child mortality and morbidity in Indonesia. This study aims to assess the multilevel determinants of continuum of care (CoC) and its contextual factors in Indonesia.

**Methods:**

Data from the 2017 Indonesian Demographic and Health Survey were analyzed. A total of 14,398 women aged 15–49 years who had live births 5 years preceding the survey were analyzed for maternal CoC, while data from 9,206 women and their children (aged < 36 months) were analyzed for maternal CoC and vaccination. Maternal CoC include antenatal care visits, safe facility delivery, and postnatal care. Vaccination includes the 10 recommended dosages of vaccination adjusted for age. Multilevel logistic regression was used to analyse variations in maternal CoC at the individual, household and community levels.

**Results:**

Our findings showed that only half of the women in our study had the complete maternal CoC, and only one-third had both the complete CoC and vaccination for their children. The odds of completing CoC and vaccination are lower among women aged < 20 years, have ≥ 4 children, have low socioeconomic status, and live in rural areas or outside Java-Bali. Pregnancy-related factors were also associated with CoC completeness.

**Conclusions for Practice:**

Targeted interventions to improve awareness and increase access that go beyond the individual factors should be developed. In addition to awareness campaigns, interventions aimed at reducing disparities across the different regions in Indonesia should be implemented to improve completeness of maternal CoC and vaccination.

**Supplementary Information:**

The online version contains supplementary material available at 10.1007/s10995-025-04110-w.

## Significance

What is already known on this subject?

What this study adds?

Several determinants of the maternal continuum of care (CoC) have been identified by previous studies.

For the outcome measures, we analyze child vaccination which had been adjusted to the 10 doses recommended by the Indonesian government and calibrated for the child’s age to provide more comprehensive insights into the continuum of care for maternal and child health. Furthermore, multilevel analyses of determinants of maternal CoC and maternal CoC and vaccination—including sociodemographic, socioeconomic, pregnancy-related, and contextual factors—offer a more nuanced understanding of the factors related to CoC. These findings can inform more targeted and specific recommendations for tailored interventions aimed at priority populations.

**Supplementary Information:**

The online version contains supplementary material available at 10.1007/s10995-025-04110-w.

## Introduction

Progress in reducing maternal and child mortality and morbidity are relatively slow in low-and-middle income countries despite increasing healthcare utilization (Vallely et al., 2023). The burden of maternal mortality is concentrated in LMICs, which account for 99% of global maternal deaths (Say et al., 2014). Moreover, children under five in LMICs are at a higher risk of dying than those in high-income countries (United Nations Inter-agency Group for Child Mortality Estimation (UN-IGME), 2023). One important strategy to address the problem is an improvement in coverage and quality of maternal and child healthcare, starting from prenatal, delivery, postnatal, through the period of newborn and child healthcare (Kumar et al., 2024; Lassi et al., 2014).

Indonesia is still among the ten highest countries for maternal and child mortality (World Health Organization, 2023), and almost half of Indonesian women reported maternal morbidity during pregnancy and/or labor (Widyaningsih et al. 2017). Additionally, compared to other low-middle income countries (LMICs) and other Southeast Asian countries, maternal healthcare utilization in Indonesia is not among the highest, with persisting problem of inequity by sociodemographic factors (Feng et al., 2023). The increasing economic inequality and geographical conditions present challenges to improving the equal distribution and quality of maternal healthcare in Indonesia (Tripathi & Singh, 2017). Therefore, comprehensive assessment of the continuum of care in maternal health as well as child health is needed, to provide information for targeted intervention.

While previous studies have assessed utilization of maternal and child healthcare independently (Efendi et al., 2019; Harapan et al., 2021; Laksono et al., 2020), this study assesses the completeness in utilization of continuum of care for maternal and child health (MCH). Hence, the study will provide valuable insights into the comprehensive provision of MCH services and help identify factors contributing to persistently high maternal mortality in Indonesia, despite significant improvements in maternal health coverage.

Previous studies in other LMICs have shown that access to maternal and child healthcare is influenced by contextual factors—such as region of residence and distance to healthcare—in addition to individual factors (Gandhi et al., 2022; Tripathi et al., 2024; Usman et al., 2022). Building on previous studies on the continuum of care (CoC) in other countries as well as in Indonesia (Andriani et al., 2021; Rammohan et al., 2024), our study will add to the understanding of contextual factors as well as regional differences associated with maternal and child CoC in Indonesia. With the diverse geographical condition in Indonesia, the inclusion of the contextual factors in a multilevel analysis can be valuable to other LMICs, particularly those with similar regional disparities. This study aims to explore the multilevel analyses of determinants of the CoC, with inclusion of pregnancy-related factors, which were under-explored in previous studies, will provide a more comprehensive insights on how to improve maternal and child health in Indonesia and other low-middle income countries with similar characteristics and challenges.

## Methods

### Settings

This study is conducted in Indonesia, an archipelago of more than 17,000 islands which impose challenges in the provision of complete maternal and child health. Maternal healthcare and vaccination in Indonesia are provided by public and private providers. Most women of lower socioeconomic status generally seek maternal and child care in the public healthcare network, which include the primary health care (PHCs) and its network (Mahmood et al., 2021). The PHCs network includes auxiliary PHCs and posyandu, an integrated health service in the community run by midwives and other healthcare providers (Mahendradhata et al., 2017). Women of higher socioeconomic status, particularly those with private health insurance, generally go to the private sectors, which include obstetricians/gynaecologists (OB/GYN), private midwives, private clinics and hospitals (Cheng et al., 2025).

Data from Indonesia Demographic and Health Surveys (IDHS) 2017, a nationally representative cross-sectional survey in Indonesia were analyzed. We included women of reproductive age (15–49 years old) who had at least one birth in the five years preceding the IDHS survey year. A total of 14,398 women were included in the analysis of the maternal continuum of care, while 9,206 children born in the three years preceding the survey (from 2014 onward) were included in the analysis of vaccination status.

### Outcome Variables

The outcome variable in this study was completion of CoC for maternal health services and vaccination. A women is considered to have complete maternal CoC when she had reported receiving all maternal health services recommended along the continuum of maternal period: at least four ANC visits, delivery attended by a skilled birth attendant (SBA) in a healthcare facility, and a minimum of two postnatal care visits (one within 2 days of delivery and another within 28 days) as recommended by the Indonesian Ministry of Health (2023). For antenatal and postnatal care, we categorized women as having received ANC or PNC when they reported to being checked by checked by healthcare professionals, either at a healthcare facility or at home. There were 1.4% of women who received ANC at home, while 47.34% of women received their PNC at home. Meanwhile, for delivery, we coded women as 1 when they were attended by healthcare professionals during delivery which was conducted at a healthcare facility. Furthermore, a woman and her child are considered as having complete CoC and vaccination if the mother received all the maternal care components, and the child received the recommended basic vaccinations for children aged 0–36 months adjusted for their age (Hardhantyo & Chuang, 2021).

### Covariates

We adopted the framework on social determinants of health by the World Health Organization (WHO) which considered individual, household, and community-level variables for healthcare utilization (World Health Organization, 2010). The individual factors include socio-demographic: women’s age at delivery, parity, marital status, employment status, level of education, and media exposure (categorized as rarely/less than once a week, and often/at least once a week). We also include pregnancy related factors, such as birth readiness, which combines six indicators: planned place of delivery, transportation arrangements, assistance during delivery, payment preparation, blood donation, and family planning. We classified birth readiness into low (prepared in 2 or less categories), moderate (prepared in 3–4 categories), and high (prepared in 5–6 categories). We also include knowledge of key pregnancy danger signs, whether last child’s pregnancy was wanted, and birth order. To assess barrier to health care, we include health insurance coverage, and distance to healthcare facilities.

Household level variables include family wealth quintiles and woman’s autonomy in healthcare decision-making. Women’s autonomy is categorised as moderate when decisions are primarily made by other people such (as husband/partner or other individuals) or made jointly. It is considered high when the woman is the primary decision-maker regarding her own healthcare.

The community-level variables include regional classifications by population density and type of residence by rural-urban status. We classified provinces into three regions based on population density: 1) Java-Bali, 2) more populated other islands (population density above the national average of 112/km^2^, but < 500/km^2^); and 3) less populated other islands (population density below the national average of 112/km^2^). List of provinces in Indonesia based on these categories is presented in Supplementary Tables. Data on population density were obtained from the Indonesian National Bureau of Statistics (Central Bureau of Statistics, 2020).

### Statistical Analysis

We present both weighted and unweighted estimates in our descriptive statistics below. We conducted two multilevel regression analyses: 1) unweighted multilevel logistic regression analysis with cluster and province as contextual factors, and 2) weighted multilevel logistic regression analysis with cluster as contextual factor (presented in appendix). A p value < 0.05 was considered as statistically significant for all analyses. All the analyses were conducted using STATA version 16.0. Ethics Research Committee, Faculty of Medicine, Universitas Sebelas Maret, Indonesia, approved the study with Ethical Clearance number: 54/UN27.06.11/KEP/EC/2022.

## Results

A total of 14,398 women were included in the analyses for maternal CoC and 9,206 women for maternal CoC and vaccination. The weighted analyses provided nationally representative estimates of women in reproductive age in Indonesia who gave birth in the 5 years preceding the survey (Table 1). Most (77.2%) women delivered their child between 20–35 years of age, with mean age at delivery of 29 years. Interestingly, only 15.1% had university education, and less than 50% were working. Approximately 60% of women reported having health insurance, and around 10% reported having big problem in accessing healthcare (Table 1).

**Table 1:** Weighted and unweighted proportion of women’s socio-demographics characteristics

Characteristics	Maternal CoC N = 14,398		Sample for Maternal CoC and Vaccination N = 9,206	
	Weighted% (95%CI)	Unweighted% (95%CI)	Weighted% (95%CI)	Unweighted% (95%CI)
Age at delivery (years)				
Mean (std. dev.)	29.0 (0.1)	29.0 (6.3)	29.0 (0.1)	29.0 (6.3)
< 20	6.4 (5.9–7.0)	6.0 (5.7–6.4)	5.9 (5.3–6.6)	5.9 (5.5–6.4)
20–35	77.2 (76.3–78.0)	77.1 (76.4–77.8)	77.5 (76.4–78.5)	77.4 (76.5–78.3)
> 35	16.4 (15.7–17.2)	16.9 (16.3–17.5)	16.6 (15.7–17.6)	16.7 (15.9–17.4)
Parity				
Mean (std. dev.)	2.1 (0.1)	2.2 (1.3)	2.1 (0.1)	2.2 (1.3)
1	35.5 (34.5–36.5)	33.0 (32.2–33.7)	34.7 (33.5–35.9)	33.3 (32.3–34.2)
2	35.9 (34.9–36.9)	34.0 (33.1–34.8)	36.4 (35.1–37.6)	33.9 (33.0–34.9)
3	18.4 (17.6–19.2)	19.5 (18.9–20.2)	18.8 (17.8–19.8)	19.6 (18.8–20.4)
4 or more	10.2 (9.6–10.8)	13.5 (13.0–14.1)	10.1 (9.4–10.9)	13.2 (12.6–14.0)
Married				
Yes	96.5 (3.2–3.8)	4.4 (4.1–4.8)	97.1 (96.6–97.4)	4.0 (3.6–4.4)
No	3.5 (96.2–96.9)	95.6 (95.2–95.9)	2.9 (2.6–3.4)	96.1 (95.6–96.4)
Working				
Yes	45.6 (44.4–46.7)	52.4 (51.6–53.2)	40.2 (38.9–41.5)	57.7 (56.7–58.7)
No	54.4 (53.3–55.6)	47.6 (46.8–48.4)	59.8 (58.5–61.1)	42.3 (41.3–43.3)
Education				
Completed primary or less	25.7 (24.4–27.0)	25.1 (24.4–25.8)	23.8 (22.4–25.2)	23.3 (22.4–24.2)
Incomplete secondary	28.9 (27.8–30.0)	26.1 (25.3–26.8)	28.9 (27.6–30.3)	26.1 (25.2–27.0)
Completed secondary	30.3 (29.2–31.5)	31.0 (30.3–31.8)	30.6 (29.3–31.9)	31.0 (30.1–32.0)
Higher	15.1 (14.2–16.1)	17.9 (17.3–18.5)	16.8 (15.7–18.0)	19.6 (18.8–20.4)
Media Exposure				
Rarely	12.4 (11.7–13.2)	14.6 (14.1–15.2)	12.9 (12.0–13.9)	15.1 (14.4–15.9)
Often	87.6 (86.8–88.3)	85.4 (84.8–85.9)	87.1 (86.1–88.0)	84.9 (84.1–85.6)
Birth Prepared				
Low	17.1 (16.1–18.1)	18.5 (17.8–19.1)	15.6 (14.6–16.7)	17.0 (16.2–17.8)
Moderate	35.3 (34.2–36.5)	37.2 (36.4–38.0)	35.3 (34.0–36.6)	37.4 (36.4–38.4)
High	47.6 (46.3–48.9)	44.4 (43.6–45.2)	49.1 (47.6–50.6)	45.6 (44.6–46.6)
Know Danger in Pregnancy				
Yes	70.1 (69.0–71.2)	66.2 (65.4–67.0)	71.2 (69.8–72.5)	67.2 (66.3–69.2)
No	29.9 (28.8–31.1)	33.8 (33.0–34.6)	28.8 (27.5–30.2)	32.8 (31.8–33.7)
Last Child Wanted				
Yes	83.9 (83.1–84.6)	83.4 (82.8–84.0)	83.6 (82.6–84.6)	83.2 (82.4–84.0)
No	16.1 (15.4–16.9)	16.6 (16.0–17.2)	16.4 (15.4–17.4)	16.8 (16.1–17.6)
Health Insurance				
Yes	59.3 (58.1–60.5)	62.7 (61.9–63.5)	62.0 (60.5–63.4)	35.2 (34.3–36.2)
No	40.7 (39.5–42.0)	37.3 (36.6–38.1)	38.0 (36.6–39.5)	64.8 (63.8–65.8)
Distance to Healthcare				
Big problem	10.6 (9.8–11.5)	11.1 (10.6–11.6)	11.1 (10.0–12.3)	11.5 (10.8–12.1)
Not a big problem	89.4 (88.5–90.2)	88.9 (88.4–89.4)	88.9 (87.8–90.0)	88.5 (87.9–89.2)
Households Wealth				
Poorest	18.3 (17.2–19.5)	24.6 (23.9–25.3)	18.4 (17.1–19.7)	24.7 (23.8–25.5)
Poorer	20.1 (19.1–21.1)	19.8 (19.2–20.5)	20.9 (19.8–22.1)	20.5 (19.6–21.3)
Middle	21.0 (20.0–22.0)	19.3 (18.7–20.0)	20.4 (19.3–21.5)	19.0 (18.2–19.9)
Richer	21.0 (20.0–22.0)	18.7 (18.0–19.3)	20.8 (19.7–22.0)	18.3 (17.5–19.1)
Richest	19.6 (18.3–20.8)	17.6 (17.0–18.3)	19.5 (18.2–21.0)	17.6 (16.8–18.4)
Women’s involvement in Decision Making				
Moderate	34.0 (32.9–35.2)	32.4 (31.6–33.1)	33.7 (32.3–35.0)	32.0 (31.0–32.9)
High	66.0 (64.8–67.1)	67.7 (66.9–68.4)	66.3 (65.0–67.7)	68.0 (67.1–68.9)
Urban				
Yes	49.3 (48.1–50.4)	50.3 (49.5–51.2)	49.3 (47.9–50.7)	50.2 (49.2–51.2)
No	50.7 (49.6–51.9)	49.7 (48.8–50.5)	50.7 (49.4–52.1)	49.8 (48.8–50.8)
Region				
Java-Bali	58.1 (57.0–59.2)	34.2 (33.4–34.9)	57.3 (56.0–58.6)	33.4 (32.5–34.4)
More populated other islands	27.0 (26.1–28.0)	34.8 (34.0–35.6)	27.6 (26.5–28.7)	35.2 (34.3–36.2)
Less populated other islands	14.9 (14.2–15.6)	31.1 (30.3–31.8)	15.2 (14.4–16.0)	31.4 (30.4–32.3)

Table 2 shows the proportion of maternal Continuum of Care (CoC Mom) and maternal Continuum of Care with child vaccination (CoC All) disaggregated by sociodemographic status. In Indonesia, 49.6% of women completed maternal CoC, however, only 30.7% of respondents continued to complete CoC through to child vaccination. Differences by sociodemographic factors were observed, with younger women and those with 4 or more children having lower proportion of completing maternal CoC (around 40% and 32% respectively). Differences by socioeconomic and region were also observed. Women with lower levels of education and those from lower socioeconomic backgrounds had lower rates of completing care from the maternal period through to child vaccination. Women who had higher score for birth preparedness, know danger signs in pregnancy, and wanted their last child were more likely to access all services in the continuum of care (Table 2).

**Table 2:** Weighted and unweighted proportion of continuum of care by sociodemographic status

Characteristics	Maternal CoC		Maternal CoC and Vaccination	
	Weighted% (95%CI)	Unweighted % (95%CI)	Weighted% (95%CI)	Unweighted % (95%CI)
Total	49.6 (48.3–51.0)	42.5 (41.7–43.3)	30.7 (29.3–32.1)	25.7 (24.8–26.6)
Age at delivery (years)*				
< 20	39.5 (35.4–43.7)	31.5 (28.5–34.7)	22.6 (18.0–28.0)	18.1 (15.1–21.6)
20–35	50.8 (49.4–52.3)	43.6 (42.7–44.6)	31.6 (30.1–33.2)	26.5 (25.5–27.5)
> 35	49.2 (46.6–51.7)	42.4 (40.5–44.4)	29.1 (26.2–32.0)	24.6 (22.5–26.8)
Parity*				
1	53.7 (51.7–55.6)	46.8 (45.4–48.2)	33.9 (31.7–36.2)	28.7 (27.2–30.4)
2	52.6 (50.7–54.5)	45.7 (44.3–47.1)	33.3 (31.2–35.5)	28.1 (26.5–29.7)
3	46.0 (43.5–48.5)	39.8 (38.0–41.6)	27.6 (24.8–30.6)	23.8 (21.8–25.8)
4 or more	31.7 (29.0–34.6)	28.1 (26.2–30.2)	16.0 (13.4–18.9)	14.7 (12.8–16.8)
Married*				
Yes	50.1 (48.7–51.4)	43.1 (42.3–44.0)	31.0 (29.6–32.5)	26.1 (25.2–27.0)
No	37.8 (33.2–42.8)	29.6 (26.2–33.3)	19.3 (14.3–25.5)	15.4 (12.0–19.5)
Working*				
Yes	52.2 (50.5–53.9)	44.6 (43.4–45.8)	32.9 (30.9–34.9)	27.9 (26.5–29.3)
No	47.5 (45.8–49.2)	40.7 (39.6–41.8)	29.2 (27.5–31.0)	24.0 (22.9–25.2)
Education*				
Completed primary or less	37.1 (34.6–39.6)	30.7 (29.2–32.3)	20.2 (17.9–22.7)	17.0 (15.4–18.6)
Incompleted secondary	48.0 (45.8–50.2)	40.5 (39.0–42.1)	31.1 (28.8–33.6)	25.5 (23.8–27.3)
Completed secondary	56.1 (54.2–58.0)	47.6 (46.1–49.1)	34.5 (32.3–36.9)	28.2 (26.5–29.8)
Higher	61.1 (58.6–63.5)	53.2 (51.2–55.1)	37.7 (34.7–40.8)	32.2 (30.1–34.4)
Media Exposure*				
Rarely	38.6 (35.6–41.7)	29.0 (27.1–31.0)	23.4 (20.4–26.8)	17.2 (15.3–19.2)
Often	51.2 (50.0–52.6)	44.8 (44.0–45.7)	31.8 (30.3–33.2)	27.2 (26.2–28.2)
Birth Prepared*				
Low	32.7 (30.2–35.3)	25.1 (23.5–26.8)	19.0 (16.4–22.0)	14.0 (12.4–15.9)
Moderate	45.0 (43.1–47.0)	38.3 (37.0–39.6)	26.5 (24.6–28.6)	21.7 (20.4–23.1)
High	59.2 (57.5–60.8)	53.4 (52.2–54.6)	37.4 (35.4–39.3)	33.2 (31.8–34.7)
Know Danger in Pregnancy*				
Yes	55.5 (54.0–57.0)	49.1 (48.1–50.1)	35.2 (33.6–36.8)	30.5 (29.3–31.6)
No	35.9 (34.0–37.9)	29.7 (28.4–31.0)	19.6 (17.6–21.7)	15.9 (14.6–17.2)
Last Child Wanted*				
Yes	51.2 (49.7–52.6)	43.8 (42.9–44.7)	31.8 (30.3–33.3)	26.5 (25.5–27.5)
No	41.5 (39.0–44.1)	36.1 (34.2–38.0)	24.9 (22.1–27.9)	21.6 (19.6–23.7)
Health Insurance*				
Yes	52.0 (50.4–53.6)	44.8 (43.8–45.8)	32.1 (30.4–33.8)	27.2 (26.0–28.3)
No	46.2 (44.3–48.0)	38.7 (37.4–40.0)	28.4 (26.4–30.5)	23.0 (21.5–24.4)
Distance to Healthcare*				
Not a big problem	51.5 (50.2–52.8)	44.3 (43.4–45.1)	32.2 (30.8–33.7)	26.9 (25.9–27.9)
Big problem	34.1 (30.6–37.7)	28.8 (26.6–31.0)	18.1 (15.2–21.5)	16.2 (14.1–18.6)
Household’s Wealth*				
Poorest	27.4 (24.5–30.6)	23.6 (22.3–25.1)	15.7 (13.4–18.3)	13.7 (12.3–15.1)
Poorer	43.7 (40.9–46.7)	37.8 (36.0–39.6)	27.7 (25.2–30.4)	22.4 (20.5–24.3)
Middle	51.4 (48.5–54.3)	45.9 (44.0–47.7)	31.8 (29.1–34.7)	28.1 (26.1–30.3)
Richer	57.6 (54.7–60.5)	52.5 (50.6–54.4)	35.4 (32.6–38.4)	32.0 (29.8–34.2)
Richest	65.9 (62.9–68.8)	60.0 (58.0–61.9)	41.6 (38.7–44.6)	37.1 (34.8–39.5)
Women’s involvement in Decision Making*				
Moderate	47.9 (45.9–50.0)	40.9 (39.5–42.3)	28.7 (26.6–30.9)	24.1 (22.6–25.7)
High	50.5 (49.0–52.1)	43.3 (42.3–44.3)	31.7 (30.0–33.4)	26.4 (25.3–27.5)
Urban*				
Yes	56.1 (54.3–57.8)	50.2 (49.0–51.3)	34.7 (32.9–36.5)	30.6 (29.2–31.9)
No	43.4 (41.4–45.4)	34.8 (33.8–35.9)	26.8 (24.8–28.9)	20.8 (19.6–21.9)
Region*				
Java-Bali	60.1 (58.1–62.1)	60.3 (59.0–61.7)	38.2 (36.0–40.3)	38.0 (36.3–39.8)
Outer Islands – more populated	39.9 (37.9–42.0)	39.1 (37.7–40.4)	22.3 (20.4–24.3)	21.3 (19.7–22.8)
More populated other islands	26.4 (24.3–28.6)	26.8 (25.5–28.1)	17.7 (15.6–20.0)	17.4 (16.0–18.8)

Less populated other islands

**Fig. 1:**
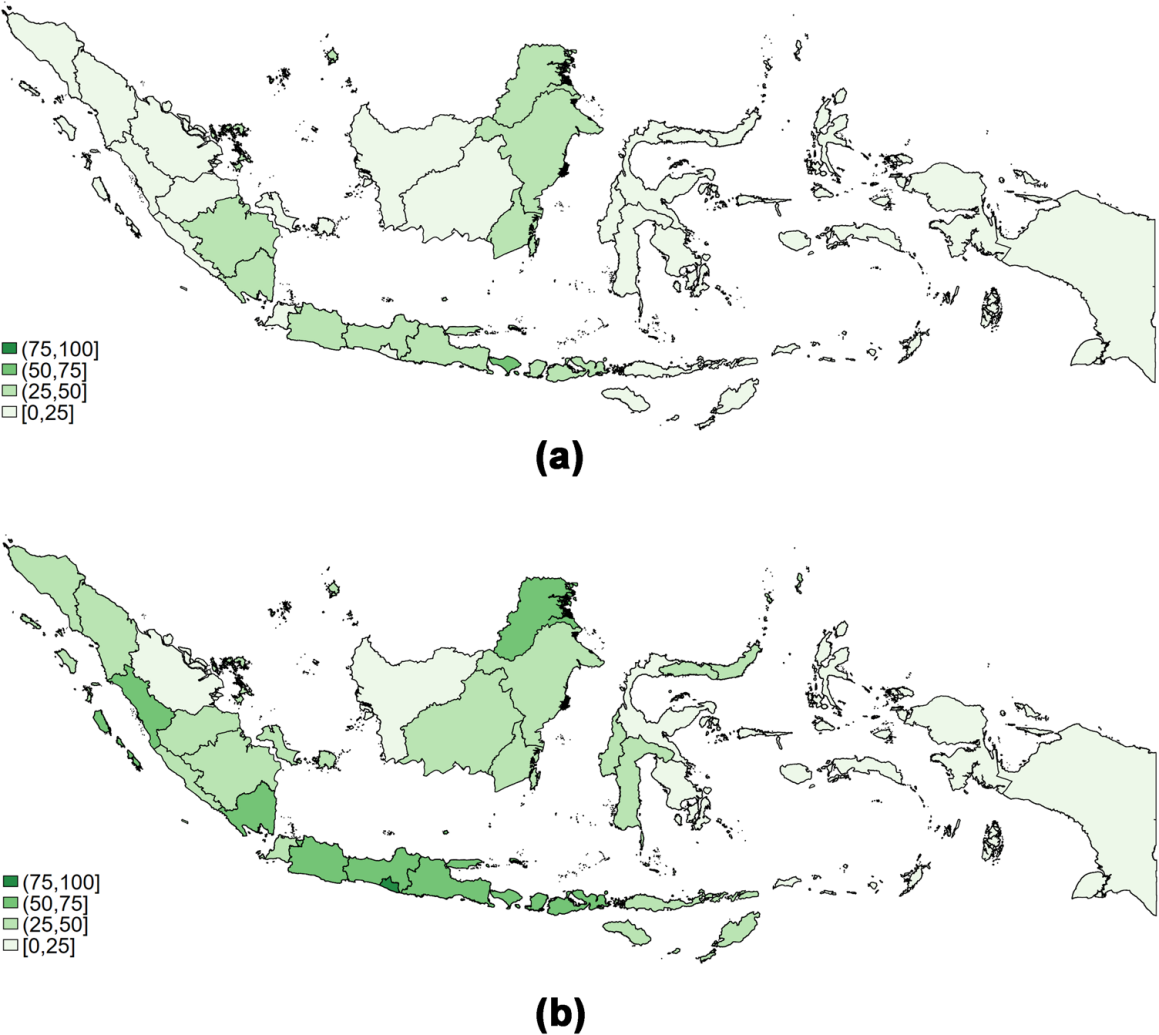
**a** Regional differences in maternal CoC. **b** Regional differences in maternal CoC and vaccination

Regional disparities remained a problem in Indonesia, with women in rural areas and those living outside of Java-Bali having significantly lower proportion of completeness of care (Table 2). Fig. 1a shows that the highest coverage of maternal CoC in Indonesia is in Java and Bali regions. Meanwhile, Fig. 1b shows the coverage of maternal CoC and vaccination in Indonesia. The figure also shows that the highest coverage is in Java and Bali regions, with the highest percentage in Bali (52.2%), followed by Central Java (48.8%) and East Java (43.3%).

### Multilevel Determinants of Continuum of Care

Table 3 shows that Model 3, which includes random effects at the province, cluster, and household levels, has a higher ICC than Model 2 (province and cluster levels) and Model 1 (province level only). The ICC for maternal CoC is 0.164 at the province level, 0.302 at the cluster-within-province level, and 0.400 at the household-within-cluster level. Similar pattern is observed for maternal CoC and vaccination. These findings highlight the importance of household- and individual- level differences in explaining variation in CoC in Indonesia. Based on the ICCs, a multilevel model with three levels of clustering were conducted and presented in Table 4, while the weighted multilevel model is presented in Supplementary Tables. There were no apparent differences in the estimates from the unweighted and weighted analyses, except for regional factors, which likely reflect the oversampling of women in the outer islands of Indonesia.

**Table 3:** Null models: Model 1 (Province level), Model 2 (Province and cluster level), and Model 3 (Province, cluster, and household level)

Characteristics	Maternal CoC			Maternal CoC and Vaccination		
	Model 1 (95% CI)	Model 2 (95% CI)	Model 3 (95% CI)	Model 1 (95% CI)	Model 2 (95% CI)	Model 3 (95% CI)
Constant	− 0.455 (− 0.720, − 0.191)	− 0.507 (− 0.803, − 0.212)	− 0.560 (− 0.892, − 0.228)	− 1.240 (− 1.462, − 1.018)	− 1.364 (− 1.609, − 1.120)	− 1.410 (− 1.754, − 1.066)
Between province variance	0.602 (0.367, 0.986)	0.740 (0.448, 1.224)	0.899 (0.518, 1.560)	0.403 (0.241, 0.675)	0.472 (0.279, 0.799)	0.501 (0.273, 0.919)
Between cluster variance	-	0.623 (0.532, 0.730)	0.758 (0.574, 1.000)	-	0.542 (0.418, 0.704)	0.578 (0.383, 0.872)
Between household variance	-	-	0.540 (0.146, 1.999)	-	-	0.175 (0.001, 32.242)
ICC (province)	0.155 (0.100, 0.231)	0.159 (0.103, 0.238)	0.164 (0.106, 0.245)	0.109 (0.068, 0.170)	0.110 (0.068, 0.172)	0.110 (0.068, 0.173)
ICC (province and cluster)	-	0.293 (0.237, 0.355)	0.302 (0.245, 0.366)	-	0.236 (0.187, 0.292)	0.237 (0.188, 0.295)
ICC (province, cluster, and household)	-	-	0.400 (0.280, 0.534)	-	-	0.276 (0.120, 0.515)
Observations	14,464			9,271		
Group level	Province	Province; cluster	Province; cluster; household	Province	Province; cluster	Province; cluster; household
Number of groups	34 provinces	34 provinces	34 provinces	34 provinces	34 provinces	34 provinces
		1,965 clusters	1,965 clusters		1,942 clusters	1,942 clusters
			14,048 household			9,059 household
Likelihood ratio test (LR)	− 9068.5	− 8850.2	− 8848.6	− 4968.6	− 4912.2	− 4912.1
Prob > chi2	(0.000)	(0.000)	(0.000)	(0.000)	(0.000)	(0.000)

Model 1: One−level model (province)

Model 2: Two−level model (province, cluster)

Model 3: Three−level model (province, cluster, household)

ICC Intraclass correlation coefficient

**Table 4:** Multilevel logistic regression of continuum of care in maternal and child: unweighted analyses

Characteristics	Maternal CoC		Maternal CoC and Vaccination	
	aOR	CI 95%	aOR	CI 95%
Age at delivery (years)*				
< 20	0.6***	0.5–0.8	0.7**	0.5–0.9
20–35	Reference	Reference	Reference	Reference
> 35	1.3***	1.2–1.5	1.2*	1.0–1.4
Parity				
1	Reference	Reference	Reference	Reference
2	0.9**	0.8–1.0	0.9	0.8–1.0
3	0.7***	0.6–0.9	0.8**	0.6–0.9
4 or more	0.5***	0.4–0.6	0.5***	0.4–0.7
Married				
No	Reference	Reference	Reference	Reference
Yes	1.2*	1.0–1.6	1.5**	1.0–2.1
Working				
No	Reference	Reference	Reference	Reference
Yes	1.1**	1.0–1.2	1.2**	1.0–1.3
Education				
Completed primary or less	Reference	Reference	Reference	Reference
Incompleted secondary	1.2***	1.0–1.4	1.4***	1.1–1.7
Completed secondary	1.4***	1.2–1.6	1.3***	1.1–1.6
Higher	1.5***	1.2–1.8	1.4**	1.1–1.7
Media Exposure				
Rarely	Reference	Reference	Reference	Reference
Often	1.4***	1.2–1.6	1.5***	1.2–1.8
Birth Prepared				
Low	Reference	Reference	Reference	Reference
Moderate	1.5***	1.3–1.7	1.4***	1.1–1.7
High	2.4***	2.0–2.8	2.1***	1.7–2.7
Know Danger in Pregnancy				
No	Reference	Reference	Reference	Reference
Yes	1.5***	1.3–1.6	1.6***	1.3–1.9
Last Child Wanted				
No	Reference	Reference	Reference	Reference
Yes	1.4***	1.3–1.6	1.2**	1.0–1.5
Health Insurance				
No	Reference	Reference	Reference	Reference
Yes	1.2***	1.1–1.3	1.3***	1.1–1.4
Distance to Healthcare				
Not a big problem	Reference	Reference	Reference	Reference
Big problem	0.7***	0.6–0.8	0.7***	0.6–0.9
Household Wealth				
Poorest	Reference	Reference	Reference	Reference
Poorer	1.3***	1.1–1.5	1.4***	1.1–1.7
Middle	1.6***	1.3–1.8	1.6***	1.3–2.0
Richer	1.8***	1.5–2.2	1.7***	1.3–2.2
Richest	2.1***	1.7–2.6	2.0***	1.5–2.6
Woman’s involvement in Decision Making				
Moderate	Reference	Reference	Reference	Reference
High	1.1	1.0–1.2	1.1	0.9–1.2
Urban				
No	Reference	Reference	Reference	Reference
Yes	1.2***	1.1–1.4	1.1	0.9–1.3
Region				
Java-Bali	Reference	Reference	Reference	Reference
More populated other islands	0.4***	0.2–0.6	0.5**	0.3–0.9
Less populated other islands	0.2***	0.1–0.4	0.5***	0.3–0.8
*ICC (province level)*	0.056	0.033–0.094	0.061	0.035–0.103
*ICC (province and cluster level)*	0.175	0.145–0.210	0.175	0.137–0.221
ICC (province, cluster, and household level)	0.253	0.139–0.414	0.293	0.133–0.528

Adjusted Odds Ratio (aOR) with 95%CI of age at delivery variable in continuous scale is 1.0 (1.0–1.0) for both maternal CoC and maternal CoC and vaccination, and both are statistically significant at 1 percent of the confidence intervals

An odds ratio is statistically significant at either 1 percent (***), 5 percent (**), or 10 percent (*) of the confidence intervals

Table 4 presents the results of a multilevel logistic regression analysis used to identify high-risk groups for not completing the continuum of care (CoC). Women who are younger (< 20 years old), had high parity (≥ 4), not married, and not working, had lower odds for completing maternal CoC as well as maternal CoC and vaccination. Furthermore, women with lower levels of education and from families in the lowest wealth quintiles also have lowest odds to complete maternal CoC and vaccination, compared to women from other groups (Table 4). Regarding pregnancy related factors, women who have more birth readiness, recognise the danger signs during pregnancy, and wanted the pregnancy have higher odds of completing maternal CoC and vaccination.

The analyses also showed the importance of both financial and physical access to healthcare. Women without health insurance and those who reported that distance to healthcare was a big problem had lower odds of completing CoC. In terms of regional factors, women living in rural areas and in outer Islands and less populated provinces in Indonesia, also had lower odds to complete CoC. The regional differences were also highlighted by the results of the multilevel analyses. A relatively higher ICC was observed at the household-within-cluster level indicating variability between households was an important factor for CoC completion, even after adjusting for individual and family-level factors included in the model. There was also significant variation in CoC at the cluster level (defined by sampling blocks in the IDHS, reflecting subdistrict-level variation). The lower ICC observed at the provincial level may be attributed to the inclusion of regional classifications in the analysis, which grouped Indonesian provinces into three categories (as shown in Table 4). To provide a more comprehensive understanding of the determinants of CoC, additional analyses are presented in the Supplementary Tables.

## Discussion

### Level of Continuum of Care

In this study, we found that only half of Indonesian women in our sample had received complete maternal continuum of care, and only a third of women and children received the complete maternal CoC and vaccination. Although the coverage of complete CoC in Indonesia is higher than African countries (Kassa et al., 2024), and slightly higher than South Asian countries such as India (Kothavale & Meher, 2021) or Pakistan (Iqbal et al., 2017), the lower rate of postnatal care utilization and vaccination in Indonesia compared to other maternal care is concerning. Previous studies in Indonesia have shown that less than half of women who received ANC and had facility delivery continued to receive PNC (Sebayang et al., 2022; Widyaningsih et al., 2022) as well as neonatal visits (Idris & Anisah, 2024). Babies who did not have PNC check-up had higher risk of having neonatal complications after birth (Kikuchi et al., 2018; Sebayang et al., 2022).

Additionally, we found that the proportion of children who received the complete ten doeses of vaccination was low. Low coverage of PNC has been shown to be associated with lower coverage of vaccination (Budu et al., 2021; Jusril et al., 2022), since women generally receive information about the importance of vaccination during PNC visits (Ntenda, 2019). These findings highlight the need to strengthen PNC services, which could potentially improve vaccination coverage.

### Determinants of Continuum of Care

Findings from this study indicate the importance of multilevel factors that significantly contribute to completion of CoC. Younger women (< 20 years old), and those with higher parity (≥ 4 children) had lower odds of completing maternal CoC and vaccination. This may be because younger women are often unprepared for the responsibilities of new motherhood and the physical changes that occur during pregnancy (Sekine & Carter, 2019). Regarding parity, new mothers are generally more reliant on healthcare providers for pregnancy-related information (Andriani et al., 2022), whereas multiparous women might rely on previous pregnancy experience and have more constraints in seeking healthcare (Gebremedhin et al., 2023; Tripathi et al., 2024). This study also showed that lower birth order increased the completion of CoC, which is in line with previous studies (Alamneh et al., 2022; Sserwanja et al., 2021). This could be because women experiencing childbirth for the first time are more likely to seek information and utilise mental health services.

Regarding socioeconomic factors, our study found that working, highly educated women from wealthier families have higher odds of completing CoC compared to other groups, which is consistent with findings from previous studies (Andriani et al., 2022; Kassa et al., 2024; Kothavale & Meher, 2021). Women with higher education and socioeconomic status (SES) generally have higher health literacy are better equipped to challenge negative social norms, which in turn leads to more informed healthcare decison-making and increases the likelihood of completing CoC. Interventions to remove financial barriers—such as promoting health insurance membership—has been shown to improve CoC among women of low SES (Alamneh et al., 2022; Kothavale & Meher, 2021). This is supported by our analysis, which found a significant association between health insurance membership and complete CoC.

Furthermore, our analyses also showed the importance of pregnancy related factors, including childbirth preparedness, knowledge of danger signs during pregnancy to improve the completion of CoC. Women who are well prepared for birth tend to have better knowledge of maternal and newborn health and have higher odds to complete CoC (Andarge et al., 2017). Moreover, those who are aware of pregnancy danger signs are also more likely to access healthcare (Kothavale & Meher, 2021; Muluneh et al., 2020). Our findings underscore the importance of improving awareness of maternal and child health to increase completeness of care, particularly among women at higher risk of incomplete CoC.

Our findings highlight the importance of mass media as a cost-effective platform to improve health literacy. Women with more mass media exposure have better knowledge about the danger signs of pregnancy and child birth (Wulandari & Laksono, 2020) which increase uptake of maternal and child health services (Andriani et al., 2022; Budu et al., 2021; Sserwanja et al., 2021). Consistent with previous findings on the importance of Women’s autonomy (Andriani et al., 2022; Iqbal et al., 2017; Sserwanja et al., 2021), our study underscores the importance of women’s involvement in healthcare decision-making, particularly during maternal period.

Regarding physical access to healthcare, we found that distance to health facilities is significantly associated with the odds of completing CoC. Access to healthcare is particularly important in regions with less healthcare facilities, including in rural areas and less populated islands in Indonesia. Our findings revealed persistent disparities in CoC completion by area of residence. Studies in other LMICs have also found differences in access to maternal CoC by area of residence (Andriani et al., 2022; Kassa et al., 2024; Rahut et al., 2024) and and region (Budu et al., 2021; Gebremedhin et al., 2023; Iqbal et al., 2017; Tripathi & Singh, 2017).

Women in rural and/or remote areas have lower odds of accessing healthcare, due to multiple barriers to maternal health services. These barriers include distance to health facility, availability and quality of healthcare resources, including doctors, midwives, and essential medicines (Fanda et al., 2024; Kurniati et al., 2024; Twineamatsiko et al., 2023). In contrast, better road infrastructure and adequate public transportation in urban and less remote areas facilitate easier access to health services. Persistent inequalities in socio-economic development across Indonesia—particularly in transportation, infrastructure, and healthcare availability—contribute to lower CoC completion rates in rural and remote regions. Moreover, interrelated challenges factors and complex problems such as socio-economic disparities and security can hinder women's access to maternal health services and result in them not being able to complete CoC.

### Study Limitations

This study has several limitations: First, this study uses a cross-sectional data and the conclusions drawn from these findings are not causal. However, the use of multilevel modelling in the analyses, facilitates understanding the association between personal and contextual factors in completeness of CoC. Second, although we limit the analyses to the last-born child within the five years preceding the survey, there is a potential for recall bias, as women may not accurately remember the maternal health services they received during that period. Third, this study analyzed data from 2017 which happened prior to COVID-19. Several studies have reported changes in maternal and child healthcare utilization in Indonesia during the COVID-19 pandemic (McGowan et al., 2023; Randell et al., 2024). However, current reports by the Ministry of Health (Indonesian Ministry of Health, 2023) showed improvement in maternal and child healthcare utilization were observed suggesting a rebound towards the pre-pandemic trends, despite a decline during the pandemic. Although conducted prior to COVID-19, IDHS 2017 can be considered one the most comprehensive data on CoC in Indonesia, with the inclusion of multilevel factors, including pregnancy-related factors. Despite these limitations, this study has several major strengths, first this study analyzes the level of the continuum of care from pregnancy to new born care, and vaccination using more specific indicators for each component of CoC. Secondly, this study uses a nationally representative sample to investigate the factors associated with CoC in Indonesia. Lastly, it provides a more comprehensive analyses of multilevel determinants of CoC with the inclusion of pregnancy-related and sociodemographic factors, with particular attention to regional variations. These strengths enable the study to offer more detailed recommendations for targeted interventions in Indonesia and other countries facing similar challenges.

## Conclusions

This study revealed suboptimal coverage of CoC in Indonesia, along with significant sociodemographic and regional disparities. Younger and multiparous women had lower odds of completing the CoC, while women who were exposed to mass media, actively involved in decision-making, better prepared for birth/delivery and aware of pregnancy danger signs had higher odds of completing it. Based on these findings, there is a need to improve awareness and knowledge of maternal and child health risks through targeted counseling for high-risk women during antenatal and postnatal care. In addition, broader strategies—such as the use of mass media—should be employed to raise awareness about pregnancy danger signs and the importance of birth preparedness. Higher-level health system intervention to improve access to CoC is important, particularly, to the less populated provinces in Indonesia with limited access to healthcare.

## Supplementary Information

Below is the link to the electronic supplementary material.

Supplementary file1 (DOCX 54 KB)
