# Supplementary material for: Exploring the Multilevel Determinants of Suboptimal Maternal and Child Continuum of Care in Indonesia
Source: Matern Child Health J. 2025 Jul 4;29(7):919–31. doi: 10.1007/s10995-025-04110-w (PMC12289808; doi:10.1007/s10995-025-04110-w)
Supplement: Supplementary file 1 — Supplementary file1 (DOCX 54 KB) [file 10995_2025_4110_MOESM1_ESM.docx]

Supplementary Tables

Supplementary Table 1. Multilevel logistic regression of continuum of care in maternal and child: weighted analyses

| **Characteristics** | **Maternal CoC** | | **Maternal CoC and Vaccination** | |
| --- | --- | --- | --- | --- |
|  | **aOR** | **CI 95%** | **aOR** | **CI 95%** |
| Age at delivery (years) | | | | |
| < 20 | 0.7*** | 0.6-0.8 | 0.7* | 0.5-1.0 |
| 20-35 | Reference | Reference | Reference | Reference |
| > 35 | 1.4*** | 1.2-1.6 | 1.2* | 1.0-1.5 |
| Parity | | | | |
| 1 | Reference | Reference | Reference | Reference |
| 2 | 0.9 | 0.8-1.0 | 0.9 | 0.8-1.1 |
| 3 | 0.7*** | 0.6-0.8 | 0.7*** | 0.5-0.8 |
| 4 or more | 0.5*** | 0.4-0.6 | 0.4*** | 0.3-0.5 |
| Married | | | | |
| No | Reference | Reference | Reference | Reference |
| Yes | 1.2 | 0.9-1.5 | 1.4 | 0.9-2.1 |
| Working | | | | |
| No | Reference | Reference | Reference | Reference |
| Yes | 1.1** | 1.0-1.2 | 1.1 | 1.0-1.3 |
| Education | | | | |
| Primary school or lower | Reference | Reference | Reference | Reference |
| Secondary school | 1.3*** | 1.1-1.5 | 1.5*** | 1.2-1.8 |
| College and higher | 1.5*** | 1.2-1.8 | 1.4** | 1.1-1.8 |
| Wealth | | | | |
| Poorest | Reference | Reference | Reference | Reference |
| Poorer | 1.4*** | 1.1-1.6 | 1.5*** | 1.2-2.0 |
| Middle | 1.6*** | 1.3-1.9 | 1.6*** | 1.2-2.0 |
| Richer | 1.9*** | 1.6-2.3 | 1.6*** | 1.2-2.1 |
| Richest | 2.1*** | 1.7-2.7 | 1.9*** | 1.4-2.6 |
| Media Exposure | | | | |
| Rarely | Reference | Reference | Reference | Reference |
| Often | 1.3*** | 1.1-1.5 | 1.2* | 1.0-1.5 |
| Decision Making | | | | |
| Moderate | Reference | Reference | Reference | Reference |
| High | 1.0 | 0.9-1.2 | 1.0 | 0.9-1.2 |
| Birth Prepared | | | | |
| Low | Reference | Reference | Reference | Reference |
| Moderate | 1.4*** | 1.2-1.7 | 1.3** | 1.0-1.6 |
| High | 2.2*** | 1.9-2.5 | 1.8*** | 1.5-2.3 |
| Know Danger in Pregnancy | | | | |
| No | Reference | Reference | Reference | Reference |
| Yes | 1.5*** | 1.3-1.7 | 1.6*** | 1.4-1.9 |
| Last Child Wanted | | | | |
| No | Reference | Reference | Reference | Reference |
| Yes | 1.5*** | 1.3-1.7 | 1.3** | 1.0-1.5 |
| Health Insurance | | | | |
| No | Reference | Reference | Reference | Reference |
| Yes | 1.2*** | 1.1-1.3 | 1.1* | 1.0-1.3 |
| Distance to Healthcare | | | | |
| Not a big problem | Reference | Reference | Reference | Reference |
| Big problem | 0.6*** | 0.5-0.8 | 0.6*** | 0.5-0.8 |
| Urban | | | | |
| No | Reference | Reference | Reference | Reference |
| Yes | 1.0 | 0.9-1.2 | 0.9 | 0.8-1.1 |
| Region | | | | |
| Java-Bali | Reference | Reference | Reference | Reference |
| Outer Islands - more populated | 0.5*** | 0.4-0.5 | 0.5*** | 0.4-0.6 |
| Out Islands - less populated | 0.3*** | 0.2-0.3 | 0.4*** | 0.3-0.5 |
| *ICC (cluster level)* | 0.163 | 0.139-0.191 | 0.147 | 0.112-0.191 |
| ^An odds ratio is statistically significant at either 1 percent (***), 5 percent (**), or 10 percent (*) of the confidence intervals.^ | | | | |

Supplementary Table 2. Region category based on population density in Indonesia

| **No.** | **Category** | **Province** |
| --- | --- | --- |
| 1 | Java-Bali (population density ≥500/km^2^) | 1. Jakarta |
|  |  | 1. West Java |
|  |  | 1. Central Java |
|  |  | 1. Yogyakarta |
|  |  | 1. East Java |
|  |  | 1. Banten |
|  |  | 1. Bali |
| 2 | Outer Islands (population density 112 to <500/km^2^) | 1. Aceh |
|  |  | 1. North Sumatera |
|  |  | 1. West Sumatera |
|  |  | 1. South Sumatera |
|  |  | 1. Lampung |
|  |  | 1. Bangka Belitung |
|  |  | 1. West Nusa Tenggara |
|  |  | 1. West Kalimantan |
|  |  | 1. South Kalimantan |
|  |  | 1. North Sulawesi |
|  |  | 1. South Sulawesi |
|  |  | 1. Gorontalo |
|  |  | 1. West Sulawesi |
| 3 | Out Islands (population density <112/km^2^) | 1. Riau |
|  |  | 1. Jambi |
|  |  | 1. Bengkulu |
|  |  | 1. Riau Islands |
|  |  | 1. East Nusa Tenggara |
|  |  | 1. Central Kalimantan |
|  |  | 1. East Kalimantan |
|  |  | 1. North Kalimantan |
|  |  | 1. Central Sulawesi |
|  |  | 1. Southeast Sulawesi |
|  |  | 1. Maluku |
|  |  | 1. North Maluku |
|  |  | 1. West Papua |
|  |  | 1. Papua |

Supplementary Table 3. Multilevel logistic regression of continuum of care in maternal health: unweighted analyses

| **Characteristics** | **ANC** | | **ANC + SBA** | | **ANC + SBA + FB** | | **ANC + SBA + FB + PNC** | |
| --- | --- | --- | --- | --- | --- | --- | --- | --- |
|  | **aOR** | **CI 95%** | **aOR** | **CI 95%** | **aOR** | **CI 95%** | **aOR** | **CI 95%** |
| Age at delivery (years) | | | | | | | | |
| < 20 | 0.4*** | 0.3-0.5 | 0.5*** | 0.4-0.6 | 0.5*** | 0.4-0.6 | 0.6*** | 0.5-0.8 |
| 20-35 | Ref | Ref | Ref | Ref | Ref | Ref | Ref | Ref |
| **> 35** | **0.9** | **0.8-1.1** | **1.1** | **0.9-1.2** | **1.2**** | **1.0-1.4** | **1.3***** | **1.2-1.5** |
| Parity | | | | | | | | |
| 1 | Ref | Ref | Ref | Ref | Ref | Ref | Ref | Ref |
| 2 | 1.0 | 0.9-1.1 | 0.9* | 0.8-1.0 | 0.8*** | 0.7-0.9 | 0.9** | 0.8-1.0 |
| 3 | 0.9* | 0.7-1.0 | 0.8*** | 0.7-0.9 | 0.7*** | 0.6-0.8 | 0.7*** | 0.6-0.9 |
| 4 or more | 0.6*** | 0.5-0.7 | 0.5*** | 0.4-0.7 | 0.5*** | 0.4-0.6 | 0.5*** | 0.4-0.6 |
| Married | | | | | | | | |
| No | Ref | Ref | Ref | Ref | Ref | Ref | Ref | Ref |
| **Yes** | **1.6***** | **1.3-2.1** | **1.5***** | **1.2-1.9** | **1.4***** | **1.1-1.7** | **1.2*** | **1.0-1.6** |
| Working | | | | | | | | |
| No | Ref | Ref | Ref | Ref | Ref | Ref | Ref | Ref |
| Yes | 1.2*** | 1.1-1.4 | 1.2*** | 1.1-1.3 | 1.1** | 1.0-1.2 | 1.1** | 1.0-1.2 |
| Education | | | | | | | | |
| Completed primary or less | Ref | Ref | Ref | Ref | Ref | Ref | Ref | Ref |
| Incompleted secondary | 1.1** | 1.0-1.3 | 1.3*** | 1.2-1.5 | 1.3*** | 1.1-1.5 | 1.2*** | 1.0-1.4 |
| Completed secondary | 1.3*** | 1.1-1.5 | 1.5*** | 1.3-1.8 | 1.7*** | 1.4-1.9 | 1.4*** | 1.2-1.6 |
| Higher | 1.3** | 1.0-1.6 | 1.6*** | 1.3-1.9 | 1.8*** | 1.5-2.2 | 1.5*** | 1.2-1.8 |
| Media Exposure | | | | | | | | |
| Rarely | Ref | Ref | Ref | Ref | Ref | Ref | Ref | Ref |
| Often | 1.3*** | 1.1-1.5 | 1.3*** | 1.1-1.5 | 1.2** | 1.0-1.3 | 1.4*** | 1.2-1.6 |
| Birth Prepared | | | | | | | | |
| Low | Ref | Ref | Ref | Ref | Ref | Ref | Ref | Ref |
| Moderate | 1.4*** | 1.2-1.6 | 1.5*** | 1.3-1.7 | 1.5*** | 1.3-1.7 | 1.5*** | 1.3-1.7 |
| **High** | **1.9***** | **1.6-2.3** | **2.2***** | **1.8-2.6** | **2.3***** | **1.9-2.7** | **2.4***** | **2.0-2.8** |
| Know Danger in Pregnancy | | | | | | | | |
| No | Ref | Ref | Ref | Ref | Ref | Ref | Ref | Ref |
| Yes | 1.4*** | 1.3-1.6 | 1.5*** | 1.3-1.6 | 1.4*** | 1.2-1.5 | 1.5*** | 1.3-1.6 |
| Last Child Wanted | | | | | | | | |
| No | Ref | Ref | Ref | Ref | Ref | Ref | Ref | Ref |
| **Yes** | **1.9***** | **1.6-2.2** | **1.9***** | **1.6-2.2** | **1.6***** | **1.4-1.9** | **1.4***** | **1.3-1.6** |
| Health Insurance | | | | | | | | |
| No | Ref | Ref | Ref | Ref | Ref | Ref | Ref | Ref |
| Yes | 1.2*** | 1.1-1.4 | 1.3*** | 1.1-1.4 | 1.4*** | 1.2-1.5 | 1.2*** | 1.1-1.3 |
| Distance to Healthcare | | | | | | | | |
| Not a big problem | Ref | Ref | Ref | Ref | Ref | Ref | Ref | Ref |
| Big problem | 0.8** | 0.7-1.0 | 0.8*** | 0.7-0.9 | 0.8** | 0.7-1.0 | 0.7*** | 0.6-0.8 |
| Household Wealth | | | | | | | | |
| Poorest | Ref | Ref | Ref | Ref | Ref | Ref | Ref | Ref |
| Poorer | 1.2** | 1.0-1.4 | 1.5*** | 1.3-1.7 | 1.5*** | 1.3-1.7 | 1.3*** | 1.1-1.5 |
| Middle | 1.4*** | 1.2-1.6 | 1.9*** | 1.6-2.3 | 1.9*** | 1.6-2.3 | 1.6*** | 1.3-1.8 |
| Richer | 1.9*** | 1.6-2.4 | 2.6*** | 2.1-3.3 | 2.4*** | 2.0-3.0 | 1.8*** | 1.5-2.2 |
| Richest | **2.6***** | **2.0-3.3** | **3.5***** | **2.7-4.6** | **3.6***** | **2.8-4.6** | **2.1***** | **1.7-2.6** |
| Woman’s involvement in Decision Making | | | | | | | | |
| Moderate | Ref | Ref | Ref | Ref | Ref | Ref | Ref | Ref |
| High | 1.0 | 0.9-1.1 | 1.0 | 0.9-1.1 | 1.0 | 0.9-1.1 | 1.1 | 1.0-1.2 |
| Urban | | | | | | | | |
| No | Ref | Ref | Ref | Ref | Ref | Ref | Ref | Ref |
| Yes | **1.0** | **0.9-1.2** | **1.1*** | **1.0-1.3** | **1.8***** | **1.5-2.1** | **1.2***** | **1.1-1.4** |
| Region | | | | | | | | |
| Java-Bali | Ref | Ref | Ref | Ref | Ref | Ref | Ref | Ref |
| Outer Islands - more Populated | 0.5*** | 0.4-0.8 | 0.6*** | 0.4-0.8 | 0.4*** | 0.2-0.7 | 0.4*** | 0.2-0.6 |
| Out Islands - less Populated | 0.4*** | 0.3-0.7 | 0.5*** | 0.3-0.7 | 0.2*** | 0.1-0.4 | 0.2*** | 0.1-0.4 |
| *ICC (province level)* | 0.138 | 0.072-0.264 | 0.137 | 0.071-0.264 | 0.302 | 0.168-0.543 | 0.248 | 0.137-0.447 |
| *ICC (province and cluster level)* | 0.198 | 0.119-0.330 | 0.314 | 0.214-0.461 | 0.793 | 0.600-1.049 | 0.523 | 0.393-0.696 |
| ICC (province, cluster, and household level) | 1.036 | 0.426-2.521 | 1.000 | 0.409-2.444 | 0.481 | 0.105-2.203 | 0.341 | 0.053-2.200 |
| ^An odds ratio is statistically significant at either 1 percent (***), 5 percent (**), or 10 percent (*) of the confidence intervals.^ | | | | | | | | |

Supplementary Table 4. Multilevel logistic regression of continuum of care in child health: unweighted analyses

| **Characteristics** | **ANC** | | **ANC + SBA** | | **ANC + SBA + FB** | | **ANC + SBA + FB + PNC** | | **ANC + SBA + FB + PNC + Vaccination** | |
| --- | --- | --- | --- | --- | --- | --- | --- | --- | --- | --- |
|  | **aOR** | **CI 95%** | **aOR** | **CI 95%** | **aOR** | **CI 95%** | **aOR** | **CI 95%** | **aOR** | **CI 95%** |
| Age at delivery (years) | | | | | | | | | | |
| < 20 | **0.4***** | **0.3-0.6** | **0.5***** | **0.3-0.6** | **0.5***** | **0.3-0.7** | **0.7***** | **0.5-0.9** | **0.7**** | **0.5-0.9** |
| 20-35 | Ref | Ref | Ref | Ref | Ref | Ref | Ref | Ref | Ref | Ref |
| > 35 | **0.9** | **0.7-1.1** | **1.0** | **0.9-1.3** | **1.2**** | **1.0-1.5** | **1.3***** | **1.1-1.6** | **1.2*** | **1.0-1.4** |
| Parity | | | | | | | | | | |
| 1 | Ref | Ref | Ref | Ref | Ref | Ref | Ref | Ref | Ref | Ref |
| 2 | 0.9 | 0.7-1.1 | 0.8** | 0.7-1.0 | 0.8*** | 0.6-0.9 | 0.8** | 0.7-1.0 | 0.9 | 0.8-1.0 |
| 3 | 0.8** | 0.6-1.0 | 0.7*** | 0.6-0.9 | 0.7*** | 0.5-0.8 | 0.7*** | 0.6-0.9 | 0.8** | 0.6-0.9 |
| 4 or more | 0.5*** | 0.4-0.7 | 0.5*** | 0.4-0.6 | 0.4*** | 0.3-0.6 | 0.5*** | 0.4-0.7 | 0.5*** | 0.4-0.7 |
| Married | | | | | | | | | | |
| No | Ref | Ref | Ref | Ref | Ref | Ref | Ref | Ref | Ref | Ref |
| Yes | **2.2***** | **1.5-3.2** | **1.8***** | **1.3-2.6** | **1.4**** | **1.0-2.1** | **1.3*** | **1.0-1.8** | **1.5**** | **1.0-2.1** |
| Working | | | | | | | | | | |
| No | Ref | Ref | Ref | Ref | Ref | Ref | Ref | Ref | Ref | Ref |
| Yes | 1.3*** | 1.1-1.5 | 1.2*** | 1.0-1.4 | 1.2** | 1.0-1.4 | 1.1 | 1.0-1.2 | 1.2** | 1.0-1.3 |
| Education | | | | | | | | | | |
| Completed primary or less | Ref | Ref | Ref | Ref | Ref | Ref | Ref | Ref | Ref | Ref |
| Incomplete secondary | 1.1 | 0.9-1.4 | 1.2** | 1.0-1.5 | 1.3*** | 1.1-1.6 | 1.2** | 1.0-1.4 | 1.4*** | 1.1-1.7 |
| Completed secondary | 1.3** | 1.0-1.6 | 1.5*** | 1.2-1.9 | 1.9*** | 1.5-2.4 | 1.3*** | 1.1-1.5 | 1.3*** | 1.1-1.6 |
| Higher | 1.2 | 0.9-1.6 | 1.5*** | 1.2-2.0 | 1.8*** | 1.4-2.4 | 1.3*** | 1.1-1.7 | 1.4** | 1.1-1.7 |
| Media Exposure | | | | | | | | | | |
| Rarely | Ref | Ref | Ref | Ref | Ref | Ref | Ref | Ref | Ref | Ref |
| Often | 1.3** | 1.0-1.6 | 1.3** | 1.1-1.6 | 1.2* | 1.0-1.5 | 1.3*** | 1.1-1.6 | 1.5*** | 1.2-1.8 |
| Birth Prepared | | | | | | | | | | |
| Low | Ref | Ref | Ref | Ref | Ref | Ref | Ref | Ref | Ref | Ref |
| Moderate | 1.4*** | 1.1-1.7 | 1.5*** | 1.2-1.9 | 1.7*** | 1.4-2.2 | 1.6*** | 1.3-1.9 | 1.4*** | 1.1-1.7 |
| High | 2.0*** | 1.6-2.6 | 2.3*** | 1.8-2.9 | 2.6*** | 2.0-3.4 | 2.4*** | 2.0-3.0 | 2.1*** | 1.7-2.7 |
| Know Danger in Pregnancy | | | | | | | | | | |
| No | Ref | Ref | Ref | Ref | Ref | Ref | Ref | Ref | Ref | Ref |
| Yes | 1.5*** | 1.3-1.8 | 1.5*** | 1.3-1.8 | 1.5*** | 1.2-1.7 | 1.6*** | 1.4-1.8 | 1.6*** | 1.4-1.9 |
| Last Child Wanted | | | | | | | | | | |
| No | Ref | Ref | Ref | Ref | Ref | Ref | Ref | Ref | Ref | Ref |
| Yes | **2.0***** | **1.6-2.6** | **1.9***** | **1.5-2.4** | **1.7***** | **1.4-2.1** | **1.3***** | **1.1-1.6** | **1.3**** | **1.0-1.5** |
| Health Insurance | | | | | | | | | | |
| No | Ref | Ref | Ref | Ref | Ref | Ref | Ref | Ref | Ref | Ref |
| Yes | 1.5*** | 1.2-1.7 | 1.5*** | 1.3-1.8 | 1.7*** | 1.4-2.0 | 1.3*** | 1.2-1.5 | 1.3*** | 1.1-1.4 |
| Distance to Healthcare | | | | | | | | | | |
| Not a big problem | Ref | Ref | Ref | Ref | Ref | Ref | Ref | Ref | Ref | Ref |
| Big problem | 0.8* | 0.7-1.0 | 0.8** | 0.6-0.9 | 0.7*** | 0.6-0.9 | 0.7*** | 0.5-0.8 | 0.7*** | 0.6-0.9 |
| Household Wealth | | | | | | | | | | |
| Poorest | Ref | Ref | Ref | Ref | Ref | Ref | Ref | Ref | Ref | Ref |
| Poorer | 1.3** | 1.0-1.6 | 1.6*** | 1.3-2.0 | 1.7*** | 1.3-2.1 | 1.4*** | 1.2-1.7 | 1.4*** | 1.1-1.7 |
| Middle | 1.5*** | 1.2-2.0 | 2.1*** | 1.6-2.8 | 2.3*** | 1.7-3.0 | 1.7*** | 1.4-2.1 | 1.6*** | 1.3-2.1 |
| Richer | 2.2*** | 1.6-3.0 | 2.9*** | 2.1-4.1 | 2.8*** | 2.0-4.0 | 1.8*** | 1.5-2.3 | 1.7*** | 1.3-2.3 |
| Richest | 3.2*** | 2.2-4.7 | 4.3*** | 2.9-6.4 | 4.8*** | 3.1-7.2 | 2.4*** | 1.8-3.1 | 2.0*** | 1.5-2.7 |
| Woman’s involvement in Decision Making | | | | | | | | | | |
| Moderate | Ref | Ref | Ref | Ref | Ref | Ref | Ref | Ref | Ref | Ref |
| High | 1.0 | 0.9-1.2 | 1.0 | 0.9-1.2 | 1.0 | 0.9-1.2 | 1.1 | 1.0-1.2 | 1.1 | 0.9-1.2 |
| Urban | | | | | | | | | | |
| No | Ref | Ref | Ref | Ref | Ref | Ref | Ref | Ref | Ref | Ref |
| Yes | 1.0 | 0.9-1.2 | 1.1 | 0.9-1.3 | 1.8*** | 1.4-2.2 | 1.1 | 1.0-1.3 | 1.1 | 0.9-1.3 |
| Region | | | | | | | | | | |
| Java-Bali | Ref | Ref | Ref | Ref | Ref | Ref | Ref | Ref | Ref | Ref |
| Outer Islands - more Populated | 0.5*** | 0.3-0.8 | 0.6*** | 0.4-0.9 | 0.4*** | 0.2-0.7 | 0.4*** | 0.3-0.7 | 0.5** | 0.3-0.9 |
| Out Islands - less Populated | 0.4*** | 0.2-0.6 | 0.4*** | 0.3-0.7 | 0.2*** | 0.1-0.4 | 0.3*** | 0.2-0.5 | 0.5*** | 0.3-0.8 |
| *ICC (province level)* | 0.144 | 0.065-0.322 | 0.142 | 0.065-0.310 | 0.397 | 0.201-0.786 | 0.242 | 0.127-0.460 | 0.286 | 0.149-0.552 |
| *ICC (province and cluster level)* | 0.285 | 0.138-0.588 | 0.446 | 0.261-0.760 | 1.067 | 0.687-1.657 | 0.530 | 0.363-0.775 | 0.546 | 0.349-0.854 |
| ICC (province, cluster, and household level) | 2.170 | 0.839-5.607 | 1.682 | 0.616-4.597 | 1.324 | 0.390-4.487 | 0.189 | 0.003-13.377 | 0.618 | 0.010-3.824 |
| ^An odds ratio is statistically significant at either 1 percent (***), 5 percent (**), or 10 percent (*) of the confidence intervals.^ | | | | | | | | | | |
